# The Efficacy of Recombinant Human Erythropoietin in Treatment Chemotherapy Induced Anemia in Children Diagnosed with a Solid Cancer

**Published:** 2014-12-10

**Authors:** AA Hiradfar, M Banihosseinian

**Affiliations:** 1 Department of Pediatric Hematology and Oncology, Tabriz of University of Medical Sciences, Iran; 2 Children Cure and Health Hospital, Tabriz of University of Medical Sciences, Iran; 3 Pediatric Health Research Center, Tabriz University of Medical Sciences, Iran; 4 Children Cancer Clinic, Children Cure and Health Hospital, Tabriz of University of Medical Sciences, Iran

**Keywords:** Anemia, Recombinant Human Erythropoietin, Chemotherapy, Solid cancer

## Abstract

**Background:**

Recombinant human erythropoietin (rHuEPO) treatment can increase hemoglobin levels and decrease transfusion requirements. This study aims to investigate how blood transfusion influences Hemoglobin levels in patients receiving rHuEPO for 12 weeks.

**Materials and Methods:**

This was a case-control study of 60 patients less than 15 years with anemia and a solid tumor in any location between February 2013 and March 2014. Median age of the patients were 6.27±0.58 years (range, 0.9-14 years). The patients were randomly assigned in two groups of rHuEPO receiving group and control group. 29 Patients in rHuEPO group received 150 IU/kg/dose rHuEPO subcutaneously, 3 times a week, for 12 weeks. The number of patients received transfusion during the treatment period was compared in the preceding 12 weeks. Also, adverse events (AE) were recorded at the 4^th^, 8^th^, and 12^th^ weeks.

**Results:**

Mean hemoglobin levels, before and after study, in rHuEPO group were 8.85±1.01 g/dl and 9.90±0.29 g/dl, respectively (p<0.001) and in control group were, 9.00±0.09 g/dl and 7.81±0.23 g/dl, respectively (p=0.25). Among 60 patients initially eligible the present study, 57 (29 in rHuEPO group and 28 in control group) completed study course.There was a significant decrease in transfusion requirements in the rHuEPO receiving group (p=0.004). 5 (17.2%) patients in the rHuEPO group needed a blood transfusion, whereas 15 (53.6%) patients needed a transfusion in the control group. rHuEPO occasioned hypertension in one patient at 4^th^ week that caused to end the treatment. All other AE were transient, which did not reoccur after the transient discontinuation of the medication (p<0.05).

**Conclusions:**

Results showed that the rHuEPO (150 IU/kg/day, 3 times a week) was effective in increasing hemoglobin levels as well as decreasing blood transfusion requirements in children with anemia following intensive chemotherapy.

## Introduction

Severe anemia requiring Red Blood Cell (RBC) transfusion is a frequent complaint of chemotherapy [1,2,3]. In children with cancer, anemia has many causes including: chemotherapy-induced bone marrow suppression, hemolysis, blood loss, and erythropoietin deficiency induced by nephrotoxicity of chemotherapy medication [2,4-7]. It is well-known that blood transfusion is associated with numerous adverse effects such as the transmission of infectious diseases (HIV, HBV, HCV, and CMV), hemolysis, and Graft-Versus-Host Disease (GVHD). 

Recombinant Human Erythropoietin (rHuEPO) has been used to reduce the need for RBC transfusion in the treatment of chemotherapy-induced anemia [8-13]. Some researchers believe that chemotherapy regimens containing cisplatin (CDDP) are more associated with chemotherapy-induced anemia through drug-induced nephrotoxicity [5,14-16]. On the other hand, other researchers believe that endogenous erythropoietin level does not significantly reduce anemia in these children, rather bone marrow response to erythropoietin is relatively reduced [8,15]. 

Maintaining a higher hemoglobin concentration during chemotherapy results in a better quality of life (QOL) and may affect survival [19-22]. RHuEPO treatment has been shown to improve QOL in patients with chemotherapy regardless of cancer type and the response to chemotherapy along with reducing the need for transfusions [17-22]. Currently, many studies support health benefits of rHuEPO in the treatment of chemotherapy-induced anemia in adult populations [6-8,23-25]. Few clinical trials have been conducted on children which can recommend a standard dosage and adequate course of treatment for rHuEPO in this age group [10-13,26-30]. Dosage and duration of treatment with recombinant human erythropoietin were different in various studies. In most clinical trials, rHuEPO has been used in the treatment of anemia in cancer children with dosage of 150 to 900 units/ kg/ dose, 3 days per week to once a week, for a course up to 12 weeks.

This study was conducted to show positive effects of rHuEPO in the treatment of chemotherapy-induced anemia in children, and reducing the need for RBC transfusion. 

## Material and Methods

**Patients**

This was a case-control study. 98 pediatric patients diagnosed with a solid tumor were treated between February 2013 and march 2014 at the Oncology Department of Tabriz children hospital in Iran. Among 98 patients with solid tumor, 60 patients who fulfilled inclusion criteria were eligible to our study. The selected patients randomly assigned into control and rHuEPO receiving groups. According to malignancy type, different chemotherapy medications were used to treat the participants including: cisplatin, vincristine, cyclophosphamide, Ifosphamide, dactinomycin, procarbazine, etoposide, vinbalstine, adriamycin, cytarabine, methotrexate, bleomycin, and lomustine. In patients with Wilm’s tumor, Rhabdomyosarcoma, and Ewing sarcoma radiotherapy was used along with chemotherapy. On weekly basis, all participating patients underwent physical examination and blood pressure control, as well as complete blood count (CBC) test. In the course of study, renal function was evaluated in patients undergoing chemotherapy containing CDDP. 

Anemia was defined as hemoglobin less than 10 g/dl for participating patients based on American Association of Clinical Oncology (ASCO) guidelines for cancer patients with chemotherapy-induced anemia [31]. 

Including criteria: Patients aged <15 years diagnosed with cyto-histological solid tumor in any location as well as anemia associated to chemotherapy. There was no initial bone marrow metastasis in any of the participating patients. Patients never treated with rHuEPO before. Participants had normal renal, liver, and lung functions. All hematologic parameters including: reticulocyte count, serum ferritin level and serum folate level, Serum vitamin B12, direct and indirect Coomb’s test, as well as arterial blood gases were normal. The Patients had no blood transfusion history over the last month.

Patients were excluded if have a active hemorrhage, Hemolysis, liver dysfunction, renal failure, or pulmonary dysfunction during study or any serious side effects of rHuEPO such as uncontrolled hypertension, deep vein thrombosis, and polycythemia in participating patients.

**Treatment protocol**

rHuEPO was given at a 150 IU/kg/dose, 3 times a week, subcutaneously (rHuEPO made by Cilag Inc (Zug, Switzerland) and marketed as EPREX) in 60 patients. If a patient had any complaints in the process of rHuEPO treatment or any of exclusion criteria, drug administration was stopped. Patients with Hb levels less than 7 g/dl were given blood transfusions based on local guidelines at Oncology Department of Tabriz Children Hospital, Iran. 

Granulocyte Colony-Stimulating Factor (G-CSF) with dose of 5µg/kg, subcutaneously, was used routinely for the significant neutropenic (defined as a neutrophil count < 1000/mm^3^) patients in both groups according to local guidelines at Oncology Department of Tabriz Children Hospital, Iran. The use of G-CSF was stopped when the neutrophil count reached to 10,000/mm^3^. At the end of the 12 weeks, the need for blood transfusion and Hb levels were compared in both groups.

**Ethical Considerations**

The written informed consent was obtained from parent or guardian of each patient who was signed in the presence of clinical investigator and a witness in the participating institution. The research protocol was approved by the Ethics committee of Pediatric Health Research Center, Tabriz University of Medical Sciences. Helsinki Declaration criteria for Ethical Guidelines for Medical Research involving Human Subjects were followed [32].

**Data collection**

All data obtained from patients were completed by the clinical investigator in the participating institution. The patient's clinical evolution, rHuEPO dose, and lab test results were (hemoglobin, white blood count as well as platelet count at treatment onset and termination) recorded weekly during. 

The last Hb level before transfusion of patients, who had transfusions during the study, was recorded and included as a final Hb level for databases analysis in both groups. Adverse events were recorded carefully for each participating patients by the clinical investigator during the study. Data analysis was performed on data from patients completing treatment.

Efficacy was measured against the study hypothesis and need for RBC transfusions during the study through comparing to the number of transfusions in the 12 weeks. Hemoglobin response during rHuEPO treatment was evaluated by calculating the difference between initial and final Hb levels. 

**Statistical analysis**

Databases were created in Excel spreadsheets and processed using SPSS version 18 statistical software program and P–values less than 0.05 were considered significant. Chi-Square test was used to compare transfusion requirements between two groups. Mann-Whitney test was used to compare Hb levels in patients treated with or without CDDP and G-CSF. We used The Wilcoxon test to determine the significance of Hb increments. 

## Results

Among 60 patients initially eligible the present study, 57 (29 in rHuEPO group and 28 in control group) completed study course and were included in the data analysis. Patients’ malignancy types are presented in Table I. 

Three patients were withdrawn by clinical investigator because of adverse effects; one was withdrawn due to uncontrolled high blood pressure at week 4 in rHuEPO group; the second one due to renal failure at week 6 in control group; and the last one due to hemolysis at week 3 in control group. 

There were 30 boys and 27 girls with median age 6.27±0.58 years (range, 0.9-14 years) (Table II). Initial mean platelets count in rHuEPO group and control group were 245,000±82,000/mm3 and 207,000±63,000/mm3, respectively (p=0.6). Initial mean absolute neutrophil count (ANC) in rHuEPO group and control group were 4350±1200/mm3 and 3860±1050/mm3, respectivelt (p=0.8). 

**Figure 1:**
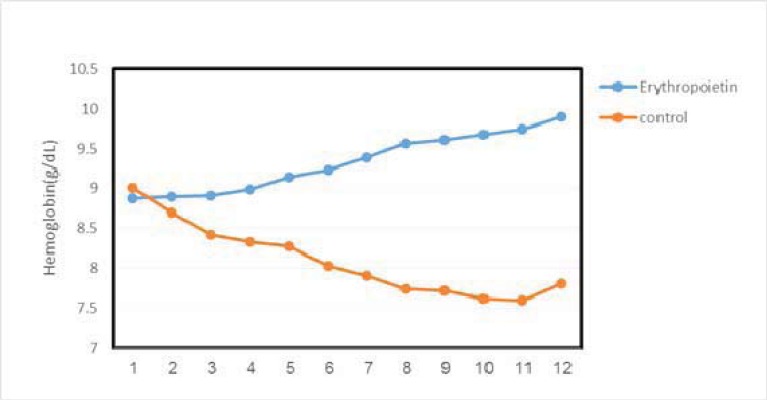
Comparison of hemoglobin ascending trend in rHuEPO and control groups who completing study course.

The mean Hb levels, before and after study, in rHuEPO group were 8.85±1.01 g/dl and 9.90±0.29 g/dl, respectively (P=<0.001). Mean Hb levels, before and after study, in control group were 9.00±0.09 g/dl and 7.81±0.23 g/dl, respectively (P=0.25). In the rHuEPO group, the increase in hemoglobin, began from the 5^th^ to 6^th^ week, and continued to the end with an ascending trend. The mean hemoglobin level was 8.90±0.10 g/dl, 8.99±0.14 g/dl, 9.23±0.24 g/dl, 9.57±0.27 g/dl, and 9.68±0.28 g/dl, and 9.90±0.29 g/dl respectively in the rHuEPO group in the 2^nd^, 4^th^, 6^th^, 8^th^, 10^th^, and 12^th^ weeks of treatment. While, it was 8.70±0.58 g/dl, 8.30±0.82 g/dl, 8.03±0.92 g/dl, 7.74±1.02 g/dl, and 7.60±1.03 g/dl, and 7.80±1.23 g/dl respectively in the control group (Figure 1).

Among the participants, 25 patients were treated with a chemotherapy protocol containing cisplatin, of whom, 11 were in rHuEPO group. At the end of the study, the difference in hemoglobin level was insignificant in patients receiving cisplatin (P=0.36). The mean initial hemoglobin levels in the rHuEPO group with or without receiving cisplatin were 8.89±0.21 g/dl and 8.83±0.11 g/dl, respectively. At the end of the study, hemoglobin levels increased to 9.67±0.49 g/dl and 10.03±0.38 g/dl, respectively in the rHuEPO group, with and without receiving cisplatin in their chemotherapy protocol (Table III). The mean initial hemoglobin levels in the rHuEPO group with or without receiving cisplatin were 8.89±0.21 g/dl and 8.83±0.11 g/dl, respectively (p=0.35). At the end of the study, hemoglobin levels increased to 9.67±0.49 g/dl and 10.03±0.38 g/dl, respectively in the rHuEPO group, with and without receiving cisplatin in their chemotherapy protocol (p=0.51). During the study, 5 patients (17.2%) in the rHuEPO group and 15 patients (53.6%) in control group, received transfusions because their hemoglobin level were below 7 g/dl. There was a significant decrease in requiring transfusions in rHuEPO group in compared to control group at the end of study (P=0.004) (Table II). Among the participants, 46 patients (80.7%) were used G-CSF to treatment 

of concurrently neutropenia during the study in both groups. 24 patients (82.4%) in rHuEPO group and 22 patients (78.5%) in control group were used G-CSF during the study. In this study, there was no significant difference in Hb level between the patients received G-CSF and those that did not use (P =0.64). The mean initial Hb levels in both groups, with or without receiving G-CSF, were 8.91±0.07 g/dl and 8.99±0.16 g/dl, respectively (p=0.66). The mean final Hb levels in the patients who received G-CSF and those that did not use it were 8.93±0.25 g/dl and 8.61±0.61 g/dl, respectively (0.64) (Table IV). Among 29 patients exposed to EPREX treatment, 35 adverse events were reported: 12 cases in weeks 1-4, 12 cases in weeks 5-8, and 11 casesin weeks 8-12. The most frequent AE was vomiting (40%), followed by fever (22.8%), bone pain (17.1%), flu-like syndrome (11.4%), flashing (5.7%), and hypertension (2.85%), (Table V). It was found no other side effects for rHuEPO treatment during the study. Considering total AE reported, only one patient was found with uncontrolled hypertension at week 4 and stopped the treatment. All other AE reported were transient, which did not reoccur after transient discontinuation of the medication.

**Table I:** Type of malignancies in participating patients

** Type of Malignancy**	**Total** **n(%)**	**rHuEPO Group** **n(%)**	**Control Group** **n(%)**	**p-value**
**Non Hodgkin lymphoma**	7(12.25)	3 (5.25)	4 (7)	0.71
**Hodgkin lymphoma**	6 (10.5)	3 (5.25)	3 (5.25)	1.00
**Epandimoma**	3 (5.25)	1 (1.75)	2 (3.5)	0.61
**Meduloblastoma **	3 (5.25)	1 (1.75)	2 (3.5)	0.61
**Neuroblastoma**	8 (14)	3 (5.25)	5 (8.75)	0.47
**Rhabdomyosarcoma **	5 (8.75)	3 (5.25)	2 (3.5)	1.00
**Ewing Sarcoma **	5 (8.75)	3 (5.25)	2 (3.5)	1.00
**Osteosarcoma **	3 (5.25)	1 (1.75)	2 (3.5)	0.61
**Synovial Sarcoma**	1 (1.75)	0 (0)	1 (1.75)	1.00
**Fibrosarcoma**	1 (1.75)	1 (1.75)	0 (0)	1.00
**Yolk Sac Tumor **	3 (5.25)	2 (3.5)	1 (1.75)	1.00
**Germinoma **	2 (3.5)	1 (1.75)	1 (1.75)	1.00
**Hepatoblastoma **	3 (5.25)	2 (3.5)	1 (1.75)	0.61
**Wilms Tumor **	6 (10.5)	4 (7)	2 (3.5)	0.67
**Nasopharyngeal Carcinoma**	1 (1.75)	1 (1.75)	0 (0)	1.00
**Total**	57	29 (50.8)	28 (49.2)	

**Table II:** Demographic and Hematologic parameters in participating patients

**Variable**			**rHEPO Group** **n=29**		**Control Group** **n=28**	**P-value**
**Gender**	Female			12(41.4%)	15 (53.6%)	0.36*
	Male			17(58.6%)	13 (46.4%)	0.36*
**Age **	Mean			6.43±0.38	6.11±0.83	0.78**
	Range			0.9-14	1-14	
**Mean Hemoglobin (g/dl)**		Onset of study		8.85±1.01	9.00±0.09	0.25**
		End of study		9.90±0.29	7.81±0.23	<0.001**
**∆ Hb (g/dl) ) end of study**				+ 1.04±0.31	- 1.31±0.24	<0.001**
**Number of Transfusion (%)**				5 (17.2%)	15 (53.6%)	0.004*

* Chi-Square Test

** Independent Samples Test

**Table III:** Patients Characteristics used CDDP in rHuEPO group & Control group

**Variable**		**rHuEPO Group**	**Control Group**	**p-value**
**Number of patients (%)**		11 (37.9%)	14 (50%)	0.36*
**Mean hemoglobin level (g/dl)**	Onset of study	8.89±0.21	9.06±0.12	0.54**
	End of study	9.67±0.49	7.71±0.36	0.001**
**∆ Hb (g/dl) at the end of study**		+ 0.78±0.59	- 1.35±0.33	0.004**
**Number of patients who had Transfusion (%)**		2 (40%)	7 (46.6%)	0.21*

* Chi-Square Test

** Mann-Whitney Test

**Table IV:** Patients Characteristics used G-CSF in the study

**Variable**		**Patients used G-CSF** **n= 46**	**Patients not used G-CSF** **n=11**	**p-value**
**Mean hemoglobin level (g/dl)**	Onset of study	8.91±0.07	8.99±0.16	0.66**
	End of study	8.93±0.25	8.61±0.61	0.64**
**∆ Hb (g/dl) at the end of study**		+0.01±0.27	-0.6±0.65	0.28**
**Number of patients who had Transfusion (%)**		4 (80%)	11 (73.3%)	0.20*

* Chi-Square Test

** Mann-Whitney Test

**Table V:** Adverse events during rHuEPO treatment*

**Adverse event**	**Weeks 1-4**	**Weeks 5-8**	**Weeks 9-12**	**Total (%)**
**Vomiting**	5(14.2%)	3(8.6%)	6(17.2)	14(40)
**Fever**	2(5.7%)	4(11.4%)	2(5.7%)	8(22.8)
**Bone pain**	1(2.9%)	3(8.6%)	2(5.7%)	6(17.2)
**Flu-like syndrome**	1(2.85%)	2(5.7%)	1(2.85%)	4(11.4)
**Flashing**	1(2.85%)	0(0%)	1(2.85%)	2(5.7)
**Hypertension**	1(2.9%)	0(0%)	0(0%)	1(2.9)
**Total**	11(31.4%)	12(34.3%)	12(34.3%)	35(100)

* Analysis of 29 patients were included in rHuEPO group

## Discussion

Anemia is a common issue in patients receiving chemotherapy, with incidence rates ranging from 51% to 74% for solid tumors and Hodgkin disease, and as high as 97% for leukemia in pediatric populations [2, 3]. Traditional therapy of chemotherapy-induced anemia was RBC transfusion, despite their short-lived effects and inherent risks [38-45]. rHuEPO treatment can improve quality of life in adult patients with chemotherapy, regardless of the disease type and the response to chemotherapy [24,33,34]. The efficacy of rHuEPO in children receiving chemotherapy is still controversial. Benefits of erythropoietin in adult treatment of chemotherapy-induced anemia were first demonstrated by Miller. Significant difference was observed in the effect of rHuEPO at dosage of 100-200 units/kg/day on increasing hemoglobin level in patients undergoing chemotherapy with and without CDDP in this study[8]. 

In a study by Porter, on 24 patients with a variety of malignant solid tumors, the use of rHuEPO of 150 units/ kg/ dose, 3 days per week, was associated with reducing the need for blood transfusion and increasing the level of hemoglobin in patients [10]. In Ragni's study on 22 patients with a variety of solid tumors, the use of rHuEPO at dosage of 150 units/ kg/ dose, 3 days per week, had positive effects on increasing patients’ hemoglobin level [12]. In both above studies, the use of recombinant human erythropoietin was also associated with reducing platelet transfusion [10, 12]. In a study by Nenadov, rHuEPO at dosage of 100 units/ kg/ dose, for two weeks, left no positive effect on the treatment of chemotherapy-inducedanemia in children [30]. In Kim's study, the use of rHuEPO at dosage of 150 units/ kg/ dose, 3 days per week, for 12 weeks was associated with reducing the need for blood transfusion in the receiving group [27]. In a study by Büyükpamukçu, on 34 patients with a variety of malignancies including solid tumors and lymphoma, the use of recombinant human erythropoietin at dosage of 150 units/ kg/ dose, 3 days per week, for 8 weeks was associated with reducing the need for blood transfusion and increasing hemoglobin in the receiving group [28].

In the present study, the use of rHuEPO at dosage of 150 units/ kg/ dose, 3 days per week, for 12 weeks in children with chemotherapy-induced anemia produced favorable outcomes such as reducing significantly (5 cases (17.2%) of blood transfusion in rHuEPO recieving group and 15 cases (53.6%) in control group) the need for blood transfusion over 12 weeks of treatment. In a few recent studies, increasing dose of rHuEPO and reducing drug injection interval produced a significant response for treating chemotherapy-induced anemia in children [24, 25, 35-37]. In Razak's study, using rHuEPO at dosage of 600-900 units/ kg/ dose, once a week, on 22 patients with a variety of non-myeloid malignancies was associated with reducing the need for blood transfusion and increasing hemoglobin level [36]. In a study by Abdelrazik and Fouda , applying rHuEPO at dosage of 450 units/ kg/ dose, once a week on 60 patients with acute lymphoblastic leukemia could increase hemoglobin in anemic children and reduce the need for blood transfusion [37]. There are a few studies on positive effects of human erythropoietin in the treatment of cisplatin-induced anemia compared to regimens without cisplatin [6, 23, 26]. In a study by Corazza, cisplatin nephrotoxicity played no significant role in decreasing erythropoietin production, while it could reduce bone marrow response to erythropoietin due to cispaltin-induced anemia. 

In the present study, no significant difference was observed in increased level of hemoglobin at dosage of 150 units/ kg/ dose, 3 days per week, for 12 weeks in patients with regimens with and without cispaltin. In this study, rHuEPO significantly increased hemoglobin level after the 5^th^ and 6^th^ weeks, and its ascending trend continued to the end of the study. In the present study, the minimum acceptable hemoglobin level was 7 g/dl. This level varied from 6 to 9 g/dl in similar studies [7, 8, 12, 28, 29, 43-45]. In this study, there was one case of hypertension in rHuEPO receiving group and all of other reported AE were transient, and did not recur after temporary discontinuation of treatment. At the end of the study, we concluded that rHuEPO at dosage of 150 units/ kg/ dose; 3 days per week can be an effective and healthy treatment for chemotherapy-induced anemia in children. 

## Conclusion

Increasing dosage of rHuEPO may be necessary in patients undergoing chemotherapy with cisplatin.

However, in the present study, similar increase in the level of hemoglobin was observed in both groups. Concurrent use of G-CSF in neutropenic patients had no effect on increasing hemoglobin level by the end of the treatment with rHuEPO.
